# Why Psychiatric Research Must Abandon Traditional Diagnostic Classification and Adopt a Fully Dimensional Scope: Two Solutions to a Persistent Problem

**DOI:** 10.3389/fpsyt.2017.00101

**Published:** 2017-06-07

**Authors:** Michael P. Hengartner, Sandrine N. Lehmann

**Affiliations:** ^1^Department of Applied Psychology, Zurich University of Applied Sciences, Zurich, Switzerland; ^2^Department of Psychiatry, Psychotherapy and Psychosomatics, University Hospital of Psychiatry, Zurich, Switzerland

**Keywords:** research, psychiatric nosology, diagnosis, psychopathology, DSM, ICD, hierarchical taxonomy of psychopathology, research domain criteria

Psychiatric research is still strongly influenced by its major classification schemes, the DSM-5 (1) and ICD-10 (2). According to these diagnostic classification systems, psychiatric nosology is construed based on multinomial taxonomic distinctions, i.e., a set of putatively independent disorder entities that are either present or absent based on polythetic-categorical criteria. Many published papers in psychiatry use these dichotomous diagnoses as the main unit of analysis. However, as it stands, these classification systems have major limitations that substantially impede scientific progress (3–5). In the following, we will detail why DSM/ICD-based approaches to the study of mental disorders are inadequate ways to address psychopathology. We will outline the major limitations of categorical psychiatric diagnoses as implemented in the current classification systems, and then we will introduce two promising approaches that were designed to circumvent these caveats.

## DSM and ICD Do Not Adequately Assess Psychopathology

No single psychiatric diagnosis has reliably shown to represent a discrete taxa, i.e., clearly demarcated disorder entities that reliably delineate disorders from each other (between-disorder boundary) and normality from disease (within-disorder boundary) (6–10). A specific example is provided below. For comprehensive reviews, see, for instance, Ref. (3, 11, 12). Most evidence suggests that psychopathology is fully dimensional by nature, but it is worth noting that there is also some inconclusive support for a few discrete taxa (7, 13). Unfortunately, current psychiatric nosology as defined by DSM/ICD does not define distinct natural entities, and diagnoses are neither mutually exclusive nor exhaustive (11, 14). Therefore, boundaries between distinct diagnoses are fuzzy, there is significant heterogeneity within diagnoses, many patients are classified with vague “not otherwise-specified” diagnoses, and comorbidity between putatively independent disorders is excessive (3, 4, 12). As it stands, psychopathology is not organized according to the DSM/ICD scheme, irrespective of whether assessed categorical or dimensional (15–19). Even the reliability of psychiatric diagnoses, once a milestone in the development of psychiatric nosology, turned out to be fairly modest or even questionable for various disorders (20, 21). Fortunately, there is an easy and straightforward solution to that problem, as dimensional representations of mental disorders have shown to substantially improve reliability and validity above and beyond categorical measures (22). In accordance, many experts in psychiatric nosology and psychopathology now claim that our current classification systems have significantly impeded further progress in etiological research and treatment development (3, 23–25).

In the following, we will briefly recapitulate some of the most pressing limitations underlying current nosology using the example of the 10 DSM-5 personality disorder (PD) diagnoses, as experts in PD research have repeatedly emphasized that these diagnostic categories are neither reliable nor valid (26–29). More specific, the 10 diagnoses listed in DSM-5 are arbitrary distinctions without empirical foundation. There is compelling evidence that PD not otherwise specified is among the most prevalent PD diagnoses despite being conceptualized as a residual category (30), which stresses the inadequate coverage of diagnostic categories. Phenotypically and genetically, personality pathology collapses into 3–5 distinct factors, but not into the 10 DSM PD diagnoses (31–34). These findings stress the problem of missing between-disorder boundaries. Contrary to prevailing diagnostic criteria, these factors are not dichotomous syndromes that are distinct from normal personality, but dimensional continua that co-vary substantially with normative personality traits (33, 35, 36). These findings stress the issue of missing within-disorder boundaries (i.e., distinction between normal and pathological). In consequence, although both DSM and ICD state that PD are enduring conditions, the categorical diagnoses that they determine are not stable at all, which stands in sharp contrast to the high stability of both normative personality and dimensional PD traits in the very same persons (37–39).

## Dimensional Models of Psychopathology

### Hierarchical Taxonomy of Psychopathology (HiTOP)

The HiTOP was developed by an independent consortium of psychopathology researchers (24). This program was a result of a long-lasting dissatisfaction with the current classification systems and their inability to adequately model the structure of psychopathology. Its major objective is to provide an empirically based, fully dimensional organization of psychopathology by subjecting current diagnoses, syndromes, and symptoms to multivariate factor-analytic procedures. Recently, various authors have significantly advanced insights in the quantitative hierarchical structure of psychopathology [(15–17, 19, 40–42); for reviews, see Ref. (43, 44)]. HiTOP posits that psychopathology is hierarchically structured, i.e., symptoms/signs (level 1) are nested within syndromes/traits (level 2) and the latter are nested within factors (level 3). Broad spectra are situated on top of the hierarchy (level 4). By this means, superordinate domains can account for the interdependence of lower-order structures such as correlated personality traits and psychopathological syndromes. Broad spectra at level 4 encompass internalizing pathology, externalizing pathology (comprising disinhibited and antagonistic), thought disorder (i.e., psychosis spectrum disorders), and detachment (i.e., pathological introversion). Subfactors of the internalizing spectrum at level 3 include sexual problems, eating pathology, fear, and distress, whereas subfactors of the externalizing spectrum include substance abuse and antisocial behavior. For more details, see Ref. (24). A pioneering innovation of the HiTOP is its incorporation of personality traits into the structure of psychopathology. This is an important step toward an evidence-based psychiatric nosology, as a compelling body of evidence has confirmed that personality substantially impacts on the occurrence, re-occurrence, and persistence of psychopathology (45–48), comorbidity between mental disorders (49–52), and, most importantly, on the liability to and course of broad internalizing and externalizing spectra (19, 53–55), and the general factor of psychopathology (40, 56–58). Though not its main target, HiTOP can also advance research on genetic vulnerabilities and genetic markers, as the proposed hierarchical structure and correlations between its dimensions appear to reflect common genetic liabilities (58–63). It is further posited that HiTOP dimensions may show clearer and stronger links to neurobiological marker, that they can effectively capture illness course, that they account for functional impairment related to psychopathology, and that they may predict efficacy and generalizability of treatments (24).

### Research Domain Criteria (RDoC)

The RDoC program was initiated by the National Institute of Mental Health (NIMH) due to the organization’s discontent with the current psychiatric classification system (64). As psychiatric diagnoses do not represent valid disease entities, the NIMH decided to allocate its resources away from the DSM and toward genetic, neuroimaging, and cognitive sciences to design a diagnostic scheme based on neurobiological and behavioral phenotypes. Despite substantial efforts during the last two decades, genetic research and the neurosciences have largely failed to detect any reliable marker that could be applied for clinical tests to aid diagnosis and prognosis (23, 65). This failure is untenable for a specialty that contemplates a future within the clinical neurosciences (66), and lack of progress was in part attributed to the constraints imposed by the invalid DSM/ICD-based diagnostic categories (3). Therefore, RDoC set out to develop a classification system that would advance insights into the neurobiology of mental disorders. This system adopts a fully dimensional and translational scheme of mental disorders, i.e., it views psychopathology in terms of deviations in fundamental functional systems. In contrast to the standard DSM/ICD top-down approach, which defines a mental disorder on the basis of signs and symptoms and then seeks to uncover the etiopathology underlying these disorders, RDoC encourages a bottom-up approach. This strategy involves that researcher define the normal distribution of a given trait or characteristic, that they examine the brain system that implements this function, and then which factors may account for dysregulation or dysfunction in these systems resulting in psychopathological symptoms and syndromes (65). The project directs research toward five neurobiological domains, specifically, negative valence systems, positive valence systems, cognitive systems, systems for social processes, and arousal/modulatory systems. These domains are divided into several units of analysis, including genes, circuits, physiology, and behavior. Each domain further contains several constructs. For instance, the negative valence systems domain involves the constructs of acute threat (fear), potential threat (anxiety), sustained threat, loss, and frustrative non-reward (23, 65). Distinct pathophysiological mechanisms subsumed under the same fuzzy diagnostic DSM/ICD-based category are seen by the pharmaceutical industry as a major cause of the low-response rate of psychiatric drugs (67). RDoC therefore constitutes a promising biological approach that could help to circumvent the problems associated with diagnostic heterogeneity and, accordingly, the lack of pathophysiological specificity within and between psychiatric diagnoses. As stated by Cuthbert and Insel (23), “As yet, the field of mental disorders research lags badly behind the rest of medicine in moving toward precision medicine” (p. 3). As a result, various pharmaceutical companies have withdrawn their investments from research on new psychiatric drugs (68). The authors of RDoC hence legitimately claim that this neurobiological classification system may substantially improve etiological research, promote the development of new treatments, and ease the detection of genetic and neurobiological markers for application in diagnostic clinical tests (23, 64, 65). Finally, it is worth noting that RDoC has been criticized (69, 70), but a detailed account is beyond the scope of this opinion paper.

## Conclusion and Outlook

HiTOP and RDoC are recently developed dimensional frameworks aimed at replacing the systematically flawed classification systems of DSM-5 and ICD-10 for research purposes. These two pioneering approaches are best seen as complementary rather than as competing systems. Both approaches have in common that they adopt a hierarchical, fully dimensional scheme. However, while HiTOP focuses exclusively on psychopathological symptoms, syndromes, and broad phenomenological domains, RDoC is largely aimed at examining genetic and neurobiological units of analysis. Therefore, RDoC holds particular promise for advancing neurobiological research relevant to psychopathology, while HiTOP is mainly concerned with restructuring psychiatric nosology by providing an empirical organization of psychopathology (24). Psychiatric research into the epidemiology of mental disorders has long struggled to accurately describe the development and course of psychopathology and how risk factors relate to the cooccurrence and stability of mental disorders due to the field’s predominant focus on inadequate, discrete diagnostic taxa (12, 44). With respect to neurobiological research, the inherent limitations of the heterogeneous and fuzzy DSM/ICD diagnoses were recently disclosed by a comprehensive meta-analysis of functional neuroimaging studies in patients with unipolar depression (71). In this work, it was demonstrated that no single pattern of aberrant brain activation consistently replicates across cognitive and emotional processing experiments. These inconsistencies may in part relate to inadequate statistical procedures and other methodological biases (72, 73), but heterogeneous clinical populations based on distinct neurobiological etiopathologies and symptom profiles are also major contributors (71). Psychiatric research must, therefore, abandon traditional DSM/ICD nosology and adopt valid and evidence-based dimensional schemes if it wants to advance insights into the etiology of mental disorders. Finally, and worthy of note, some have suggested that DSM/ICD-based diagnoses may have clinical utility despite their poor validity (11, 17). However, how could a psychiatric nosology have clinical utility when it impedes the development of efficacious treatments and clinical tests, an improved understanding of etiology and a reliable prediction of illness course (26, 27, 66)? Moreover, critics of dimensional approaches typically stress that clinical practice requires binary diagnoses. However, at this stage, both HiTOP and RDoC are merely research programs, so diagnostic entities are not necessary (3, 23, 24). Once dimensional concepts are ready for implementation in clinical practice, one could easily transform dimensional scores into categorical distinctions, just like we treat intellectual disabilities or how Peter Tyrer and the ICD-11 PD work group conceptualized the continuum of PD severity, comprising no personality disturbance, personality difficulties, mild PD, moderate PD, and severe PD (28). Such a categorical gradation considers that psychopathology is dimensional by nature but at the same time allows for making diagnoses in order to identify people who require treatment or disability benefits.

## Author Contributions

MH drafted the manuscript. SL contributed in writing and critical revision.

## Conflict of Interest Statement

The authors declare that the research was conducted in the absence of any commercial or financial relationships that could be construed as a potential conflict of interest.
